# Nucleoside-Lipid-Based Nanocarriers for Sorafenib Delivery

**DOI:** 10.1186/s11671-017-2420-2

**Published:** 2018-01-11

**Authors:** Sebastien Benizri, Ludivine Ferey, Bruno Alies, Naila Mebarek, Gaelle Vacher, Ananda Appavoo, Cathy Staedel, Karen Gaudin, Philippe Barthélémy

**Affiliations:** 1 University of Bordeaux, ARNA laboratory, F-33000 Bordeaux, France; 2 INSERM, U1212, ARNA laboratory, F-33000 Bordeaux, France; 3 CNRS, UMR 5320, ARNA laboratory, F-33000 Bordeaux, France; 4 ARNA Laboratory, team ChemBioPharm, U1212 INSERM–UMR 5320 CNRS, 146 rue Léo Saignat, 33076 Bordeaux Cedex, France

**Keywords:** Sorafenib, Solid lipid nanoparticles, Nucleolipids, Hepatocarcinoma, Breast carcinoma, Luminal B

## Abstract

Although the application of sorafenib, a small inhibitor of tyrosine protein kinases, to cancer treatments remains a worldwide option in chemotherapy, novel strategies are needed to address the low water solubility (< 5 μM), toxicity, and side effects issues of this drug. In this context, the use of nanocarriers is currently investigated in order to overcome these drawbacks. In this contribution, we report a new type of sorafenib-based nanoparticles stabilized by hybrid nucleoside-lipids. The solid lipid nanoparticles (SLNs) showed negative or positive zeta potential values depending on the nucleoside-lipid charge. Transmission electron microscopy of sorafenib-loaded SLNs revealed parallelepiped nanoparticles of about 200 nm. Biological studies achieved on four different cell lines, including liver and breast cancers, revealed enhanced anticancer activities of Sorafenib-based SLNs compared to the free drug. Importantly, contrast phase microscopy images recorded after incubation of cancer cells in the presence of SLNs at high concentration in sorafenib (> 80 μM) revealed a total cancer cell death in all cases. These results highlight the potential of nucleoside-lipid-based SLNs as drug delivery systems.

**Electronic supplementary material:**

The online version of this article (10.1186/s11671-017-2420-2) contains supplementary material, which is available to authorized users.

## Background

Sorafenib commercialized under the name of Nexavar™ is a hydrophobic drug kinase inhibitor [1] approved for the treatment of different human cancers, including advanced renal cell carcinoma (RCC) [2], hepatocellular carcinoma (HCC) [3], and advanced thyroid carcinoma. Sorafenib has multiple known protein kinase targets, including transmembrane receptors and intracellular tyrosine and serine-threonine kinases, and has also been shown to induce apoptosis. In terms of mechanism of action, sorafenib is reported to inhibit tumor growth via multi targets, acting directly on the tumor and/or on tumor angiogenesis (through inhibition of VEGFR and PDGFR signaling) [4, 5]. Its efficacy in inhibiting malignant cells growth has been demonstrated in many histological cancer types such as melanoma [6], thyroid [7], pancreatic [5], hepatocellular carcinoma, and leukemia [8], for example. However, the low water solubility, the toxicity, and side effects limit the use of sorafenib in many clinical applications. To address these issues, several sorafenib formulations are currently under investigation [9, 10], including liquid crystalline nanoparticles [11], nanoemulsion [12], cyclodextrin-modified porous silicon nanoparticles [13], drug-eluting nanocomposites [14], polyelectrolyte-based nanoparticles [15], or curcumin self-assembled nanoparticles [16]. However, lipid nanoparticles (LNs) loaded with sorafenib have been poorly investigated [17, 18].

**Fig. 1:**
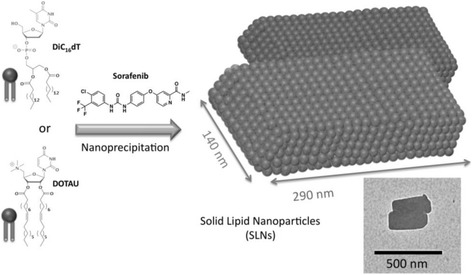
Scheme of SLNs formulation. Chemical structures of an anionic nucleotide-lipid, the thymidine 3′-(1,2-dipalmitoyl-*sn*-glycero-3-phosphate) (diC_16_dT), a cationic-nucleoside-lipid DOTAU (2′,3′-dioleyl-5′-deoxy-5′-trimethyl-ammonium-uridine), and sorafenib used in this study (left). Schematic drawing of SLNs with parallelepiped shapes obtained after nanoprecipitation of a nucleolipid (either diC_16_dT or DOTAU, leading to SLN^−^ and SLN^+^, respectively) with sorafenib (right). The schematic representation is adapted from the transmission electronic microscopy (TEM) image showing DOTAU sorafenib-loaded nanoparticles (inset, bar 500 nm)

Herein, we report the first example of sorafenib-based solid lipid nanoparticles (SLNs) [19] stabilized by nucleoside-lipids [20–22]. Chromatographic studies achieved on positively and negatively charged nucleolipids (DOTAU and diC_16_dT, respectively) indicate that these amphiphiles possess the requested stability and purity for their use within the frame of drug delivery applications [23, 24]. A simple nanoprecipitation procedure allows the formation of SLNs featuring either positive (SLN^+^) or negative charges (SLN^−^) (Fig. 1). It is worth noting that all the investigated SLNs enhanced the cytotoxic effect of sorafenib on different human carcinoma, demonstrating that SLNs can overcome the limitations of sorafenib in terms of aqueous solubility and anticancer activities.

## Methods

### Chemicals and Reagents

Methanol (MeOH), formic acid (FA), and ammonium formate (AFNH_4_) were purchased from VWR Chemicals (France) and were all HPLC (high-performance liquid chromatography) grade. HPLC grade water (minimum resistivity of 18.2 MΩ) was produced in-house by ELGA Millipore system (France). DOTAU (CAS Number: 868226-06-6) and diC_16_dT (CAS Number: 1160002-70-9) were synthesized in the lab according to the protocol reported in References [23–25]. Sorafenib, 4-[4-[[4-chloro-3-(trifluoromethyl)phenyl]carbamoy-lamino]phenoxy]-*N*-methyl-pyridine-2-carboxamide (CAS number: 284461-73-0) was purchased from SynVec http://synvec.fr (Ref# D114250414).

### Chromatography Studies

A reversed-phase UHPLC (ultra high-perfomance liquid chromatography) method was developed for nucleolipid (DOTAU and diC_16_dT) and sorafenib quantitation in SLNs. Before injection in HPLC, aqueous solutions of nanoparticles were diluted with ethanol by factors 5 and 10, to quantify nucleolipids and Sorafenib, respectively.

A chromatographic system UHPLC UltiMate 3000 from Dionex-Thermo Scientific (USA), composed of a pump with a quaternary valve system for column selection, a thermostated auto-sampler, and a thermostated column compartment, was used. The separation was carried out with the column Syncronis C18 50 × 2.1 mm, 1.7 μm. The mobile phase consisted of 70/30 MeOH/25 mM ammonium acetate (pH = 7.4) (A) and 26.5 mM ammonium acetate in MeOH (pH = 7.9) (B). A flow rate of 0.2 mL/min was used, and the gradient profile was 0–2 min, 0–100% B; 2–20 min, 100% B. The column temperature was set at 25 °C. The detection was performed at 267 nm for sorafenib and diC_16_dT and 257 nm for DOTAU. The injected volume was 1 μL leading to quantitation limits of 0.6 ng for sorafenib and 15 ng for both nucleolipids and DOTAU and diC_16_dT.

Standard curves for sorafenib, DOTAU, and diC_16_dT in ethanol are shown in Additional file 1: Figures SI1, SI2, and SI3, respectively.

### Preparation of Sorafenib Nanoparticles

Ten milligrams of sorafenib was dissolved in 1 mL of ethanol, and 10 mg of NLs (either diC_16_dT or DOTAU) was solubilized in 1 mL of ethanol. One hundred microliters of NLs, 100 μL of sorafenib solutions, and 800 μL of ethanol were mixed together at room temperature and added dropwise into 10 mL of distilled water under magnetic stirring. The solution was placed in ultrasonic bath for 90 min at 25 °C. Ethanol was removed under vacuum at 30 °C, and the volume was adjusted at 1 mL. This solution was sonicated twice by using an ultrasonic probe of 6 mm (Vibracell 75186) for 10 min at 100% of amplitude with pulse of 2 sec every 5 sec. One milliliter was dialyzed against 30 mL of distilled water for 3 × 15 min. This volume is adjusted at 2 mL and conserved for characterization quantification and stability studies. Also, control experiments were realized with the same protocol in the absence of nucleolipids.

### Transmission Electronic Microscopy (TEM and EDX)

Nanoparticles were visualized by negative staining microscopy. Ten microliters of nanoparticles was transferred to a carbon-coated copper grid for 10 min. The sample was then dried and stained with 2.5% (*w*/*w*) of uranyl acetate in water for 2 min. The specimens were observed with a Hitachi H 7650 electron microscope. Energy-dispersive X-ray spectroscopy was performed using a TECNAI transmission electron microscope coupled with Quantax-X-Flash SVE 6.

### Particle Size and Zeta Determination

Particle zeta and size were determined using a Zetasizer NanoZS, Malvern. Experiments were realized with 40 μL of the nanoparticles diluted in 400 μL of water, and measurements were performed at 25 °C.

### Cytotoxicity Analysis

HuH7 and HepG2 were grown in monolayer in DMEM-Glutamax supplemented with 10% fetal calf serum. MDA-MB-134 and T-47D were grown in monolayer in RPMI supplemented with 10% fetal calf serum (for T-47D only, nonessential AA 1X, glucose 0.45%, insulin 10 mg L^−1^, and sodium pyruvate 1X). All culture reagents were from Invitrogen. 10^4^ cells/well in 90 μL of complete culture medium were plated in a 96-well plate and incubated for 24 h at 37 °C and 5% CO_2_, before adding 10 μL of either SLN or sorafenib in the culture medium. Sorafenib (CAS number: 284461-73-0) was dissolved in culture medium with 0.1% DMSO. Note that in these conditions, the maximum concentration of sorafenib without precipitation was 5 μM, whereas for SLNs loaded with Sorafenib, the maximum concentration tested was 120 μM without DMSO. After 4 days of incubation, cell viability was assessed with the formazan-based proliferation assay (CellTiter 96® Aqueous One Solution Cell Proliferation Assay kit, Promega), by adding 20 μL/well of reagent solution. After a 30-min incubation at 37 °C, 5% CO_2_, the absorbance of each well was measured at a wavelength of 492 nm using a Berthold spectrophotometer. Results are expressed as the percentage of $\frac{{\mathrm{OD}}_{492}\ \mathrm{of}\  \mathrm{treated}\  \mathrm{cells}-{\mathrm{OD}}_{492}\ \mathrm{of}\  \mathrm{blank}}{\ {\mathrm{OD}}_{492}\mathrm{of}\  \mathrm{untreated}\  \mathrm{cells}-{\mathrm{OD}}_{492}\ \mathrm{of}\  \mathrm{blank}}$.

### Cell Viability Studies

HuH7 were grown as previously described in the “Cytotoxicity Analysis” section. The cell viability was performed after 4 days of incubation with SLN^+^ loaded with sorafenib at different concentrations (0, 1, 5, 10, 25, 50, and 100 μM) using live/dead cell viability assay (Invitrogen). Briefly, the culture medium was removed, and adherent cells were washed once with Hanks’ Balanced Salt Solution (HBSS). Two hundred microliters of HBSS containing 2 μM calcein acetoxymethyl ester and 4 μM ethidium homodimer-1 was added to each well and incubated for 45 min at 37 °C and 5% CO_2_. After staining, the cells were washed once with HBSS and microscopically imaged on an inverted fluorescence microscope. The percentage of dead cells was assessed by fluorescence-activated cell sorting (FACS) analysis. After staining with 4 μM of ethidium homodimer-1 solution, cells were treated with trypsin and washed twice with phosphate-buffered saline (PBS) by centrifugation at 1000 rpm for 5 min. Data were acquired on LSRFortessa flow cytometer from Becton Dickinson, and the results were analyzed using DIVA software. A sample of dead cells was prepared using 70% methanol and used as a control.

## Results and Discussion

### Synthesis and Characterization of SLNs

The non-toxicity and the self-assembly properties of the nucleolipids render them ideal amphiphilic adjuvants for encapsulating hydrophobic drugs such as sorafenib. In this study, a simple nanoprecipitation procedure was developed to address the low water solubility properties of sorafenib and enhance its anticancer activity, which limits in many cases its clinical use. Regarding the water solubility, we hypothesized that the hydrophobic character, the heterocycles, and hydrogen bonding functions of sorafenib would favor the interactions with nucleolipids and the formation of nanoobjects. Also, it was expected that SLNs loaded with sorafenib should increase the antitumor activities by enhancing the cellular uptake of sorafenib. Indeed, in one of our previous study achieved on cisplatin nanoparticles, we demonstrated that the enhancement of antitumor activities of cisplatin was due to an increased amount of drug internalized into cancer cells [23] [1]. Our nanoprecipitation process involves three steps: (i) the solubilization of sorafenib in ethanol at 40 °C (10 mg/mL of sorafenib, 100 μL) and addition of one equivalent of nucleolipid (either an anionic nucleotide-lipid, the diC_16_-3′-dT [thymidine 3′-(1,2-dipalmitoyl-*sn*-glycero-3-phosphate)] or a cationic nucleoside-lipid DOTAU [23] [2′,3′-dioleyl-*5′*-deoxy-5′-trimethyl-ammonium-uridine], 100 μL of a solution at 10 mg/mL in ethanol; (ii) 1 mL of the ethanol solution are added dropwise at room temperature to 10 mL of distilled water; and (iii) the resulting suspension was evaporated to remove the excess of ethanol and sonicated.

### Chromatographic Studies

To evaluate the drug loading capabilities of the novel formulations, a reversed-phase UHPLC method was developed. Using this HPLC method, the simultaneous separation of sorafenib and nucleolipids was achieved in 12 min, allowing the individual quantization of compounds in SLNs (Additional file 1: Figure SI1–4).

Loading ratios (mass ratios of sorafenib/nucleolipids in nanoparticles) of 50 and 80% and encapsulation yields of sorafenib around 55 and 75% were obtained for formulations composed of sorafenib/DOTAU (SLN^+^) and sorafenib/diC_16_dT (SLN^−^), respectively. During the control experiment performed in the absence of nucleolipid, about 90% of sorafenib was lost during the different steps of the formulation probably due to the low solubility of sorafenib in water. This result demonstrates that nucleolipids are necessary to solubilize sorafenib and stabilize the SLNs.

### Physicochemical Studies

**Fig. 2:**
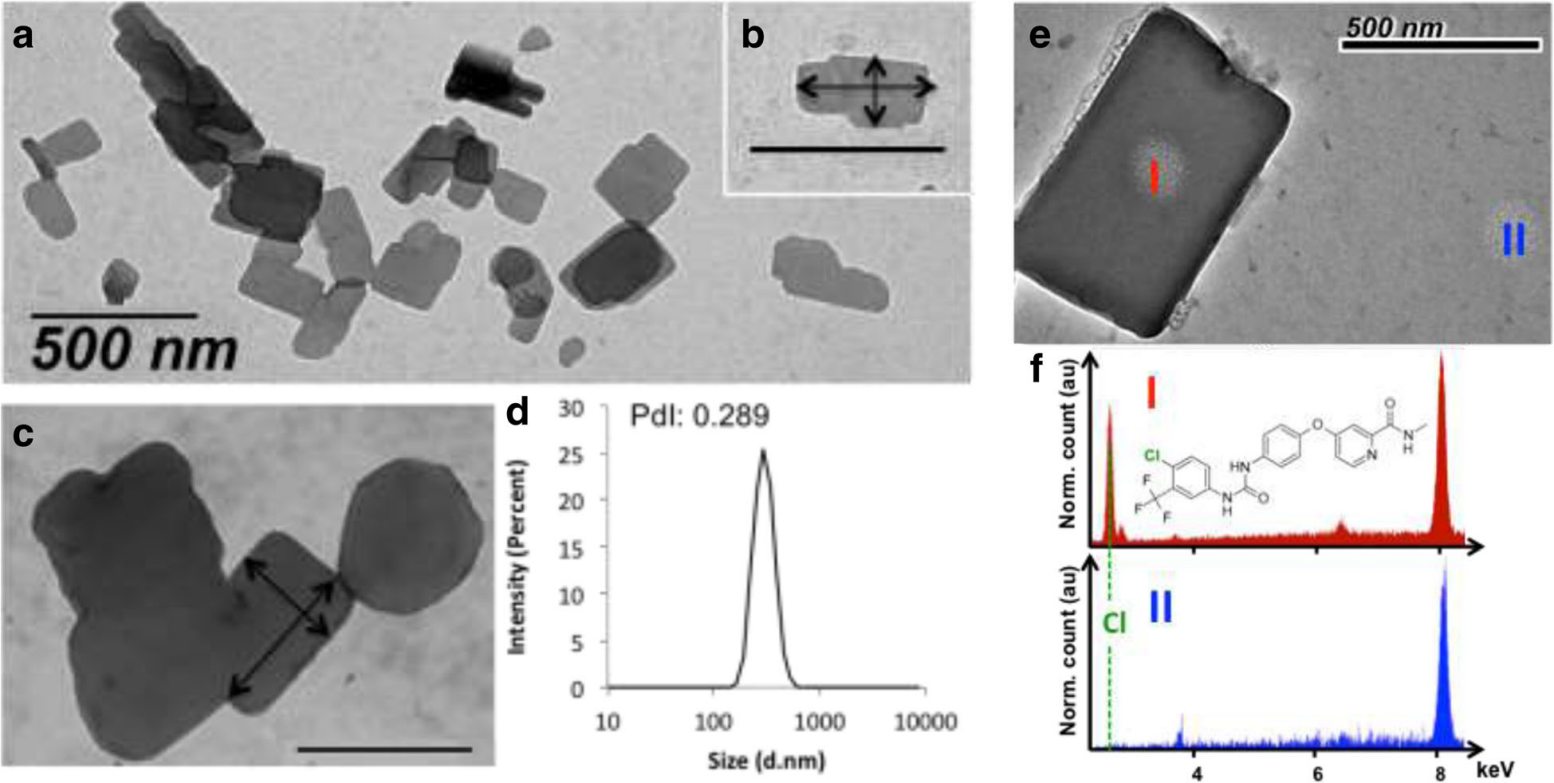
SLNs characterization. Transmission electron microscopy (TEM) images showing the morphology of sorafenib nanoparticles with DOTAU (**a**) and inset (**b**). Example of a DOTAU-based SLN featuring a size of 327 by 172 nm (arrows), which confirm an average size of 304 nm as measured by DLS (**d**). **c** Example of TEM image showing diC_16_dT-SLNs (arrows 330 by 500 nm, respectively). (**e**) TEM image of a SLN-. I & II spots are the localisations where EDX acquisitions was performed. (**f**) EDX spectra at I & II positions. Dashed line emphasized the emission of chlorine atom, which is only present in I. Chemical structure of Sorafenib molecule is also presented. Both spectra were normalized with Cu atom emission at 8 keV (due to TEM Cu grid)

Dynamic light scattering (DLS) experiments were carried out to characterize the formation of SLNs. Both negative and positive nucleolipids (diC_16_dT and DOTAU) form similar non-spherical nanoparticles with parallelepiped shapes in aqueous solution with a monodispersity (polydispersity index, PDI = 0.202 and 0.289; size = 335.2 and 304.4 nm, respectively, Fig. 2 and Additional file 1: Figure SI7). As expected, the zeta potentials of SLN-based objects depend on the nucleolipid polar heads (*ζ* = + 59.1 and − 54.9 mV for SLN^+^ and SLN^−^, see Additional file 1: Figure SI10). The presence of sorafenib in the NP was validated by TEM imaging and EDX acquisitions of a SLN− were realized with EDX acquisitions. Fig. 2e, f shows I and II spots. Spot I in Fig. 2f exhibits the emission of chlorine atom (spot I, Fig. 2e, f) indicating the presence of the sorafenib (see chemical structure Fig. 2f). Note that chlorine is present only in spot I and not in spot II (Fig. 2f).

Importantly, in control experiments, the nanopreciptation of sorafenib in the absence of nucleolipid did not give rise to any nanoobject, demonstrating that the nucleolipids allow the formation and stabilization of the SLNs.

### Stability Studies

**Fig. 3:**
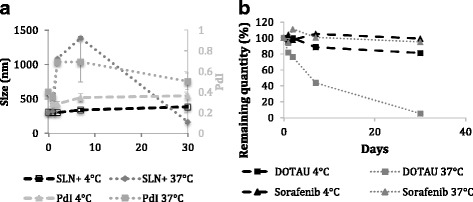
Stability studies of SLNs. Colloidal stability of SLN^+^ (**a**) and chemical stability of sorafenib and DOTAU in SLN^+^ versus time at 4 and 37 °C (**b**). PDI, polydispersity index

Colloidal stabilities of SLN^+^ and SLN^−^ were measured by DLS and zeta potential at two temperatures (Fig. 3a and Additional file 1: Figure SI10). In the case of diC_16_dT-based formulations, the size of SLN^−^ was not modified after 30 days at both 4 and 37 °C indicating a high stability of those nanoparticles (Additional file 1: Figure SI9). Such stability can be explained by the nature of the interactions (involving H-bonds, *π*-*π* stacking, charge/charge interaction) occurring between the diC_16_dT and sorafenib. However, for DOTAU-based formulations, SLN^+^ were stable only at 4 °C for 30 days as revealed by DLS studies (Fig. 3a), whereas at 37 °C, an increase of both size and PDI was observed (Fig. 3a). This relative instability observed at 37 °C in the case of SLN^+^ could be explained by repulsive coulombic interactions occurring between the positive charges of both sorafenib and DOTAU. Interestingly, the colloidal stability studies indicate that it is possible to modulate the stability, hence the delivery of sorafenib from the SLNs to the physiological environment, depending on the nucleolipid used for the stabilization of the SLNs. The stability modulation could be attractive depending on the kinetic of release needed.

In parallel to colloidal stability, the chemical stability of sorafenib, DOTAU, and diC_16_dT in the SLN-based formulations was investigated as a function of time at 4 and 37 °C using a new chromatographic method (see Additional file 1: Figure SI4). Kinetic studies at both temperatures are shown in Fig. 3b and Additional file 1: SI5 for DOTAU-SLNs and diC_16_dT-SLNs, respectively. The results show that sorafenib and diC_16_dT molecules in formulations remain stable over at least 1 month, indicating a long-term chemical stability at both temperatures in the case of SLN^−^. However, a decrease of DOTAU during the time was observed in the SLN^+^ formulations. First, at 4 °C, losses of about 12% over 7 days and 20% over 30 days were measured. When increasing the temperature, DOTAU stability was decreased with a loss of 55% over 7 days and until 95% over 30 days at 37 °C. Such differences in chemical stability between nucleolipids were already evidenced during previous stability studies (see Additional file 1: Figure SI6).

### Biological Studies

**Fig. 4:**
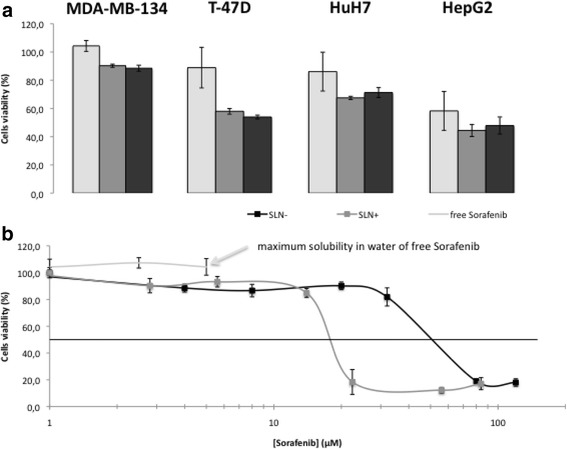
Cytotoxicity effect of sorafenib or SLNs. **a**) Comparison of the cytotoxicity with free Sorafenib or SLNs of Sorafenib in 4 cells lines (2 luminal B breast cancer and 2 hepatocarcinomas) after quantification with MTS assay on 3 wells at 5 μM of free Sorafenib, 2.8 μM of NPs Sorafenib/DOTAU (SLN+) and 4 μM of NPs Sorafenib/diC16dT (SLN-). **b**) Cell viability assay (MDA-MB-134 cells) in the presence of free sorafenib (limited of solubility ), SLN+ (grey) or SLN- (black)

**Fig. 5:**
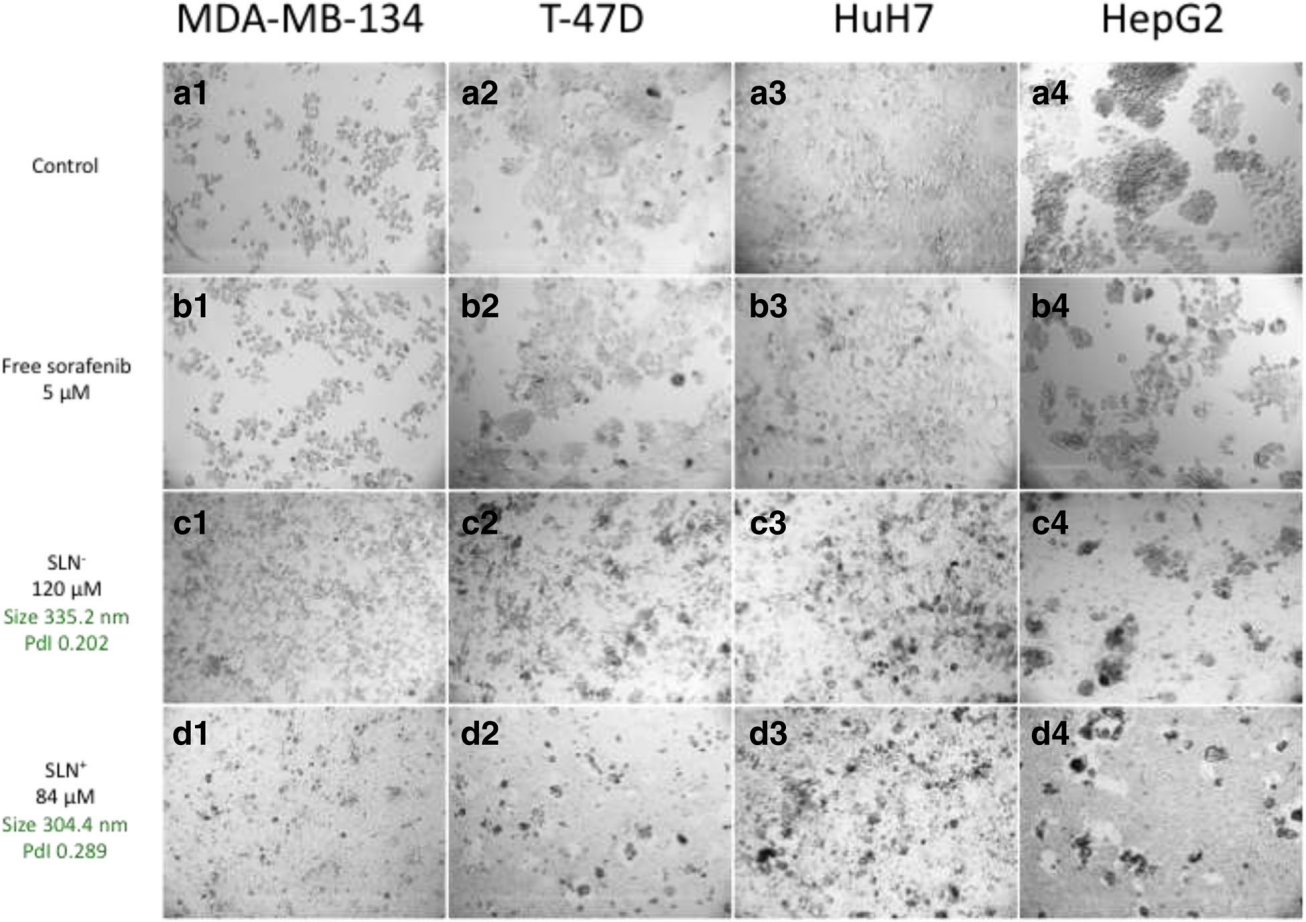
Comparison of cells morphologies between control, free sorafenib, or SLNs. Phase contrast microscopy images showing cytotoxicity in different conditions on four human carcinoma cell lines (the hepatocarcinomas, HuH7, HepG2, and the luminal breast carcinoma MDA-MB-134, T-47D). A) In the absence of sorafenib (control experiments, **A1**–**A4** for MDA-MB-134, T-47D, HuH7, HepG2, cell lines, respectively). B) Cells were incubated for 4 days in the presence of 5 μM of free sorafenib. C and D) cells incubated in the presence of SLN^−^ and SLN^+^ at 84 and 120 μM in concentration of sorafenib, respectively

SLN cytotoxicity was evaluated by metabolism activities and morphologies of cells. MTT assay allowed comparing free sorafenib (in 0.1% of DMSO) and SLNs loaded with the drug (Fig. 4) on four cell lines including two hepatocarcinomas (HuH7 and HepG2) and two luminal breast cancers (MDA-MB-134 and T-47D). First, at concentrations close to the maximum solubility of free sorafenib (5 μM of sorafenib in 0.1% of DMSO, 2.8 μM for sorafenib/DOTAU, and 4 μM for sorafenib/diC_16_dT nanoparticles), both SLNs inhibited cell viability better than free sorafenib. As shown in Fig. 4b, a cell viability study realized on MDA-MB-134 cells indicated that the activity of free sorafenib is limited by its water solubility (100% of cell viability at [sorafenib] = 5 μM), whereas IC_50_ values of 15 and 50 μM were observed for SLN^+^ and SLN^−^, respectively (Fig. 4b). In a FACS study achieved on HuH7, an IC_50_ of roughly 50 μM was obtained (Additional file 1: Figures SI12 and SI13). Images of phase contrast microscopy show indeed a reduced density of the cell layers treated with either SLN^−^ and SLN^+^ compared to untreated or sorafenib-treated cells with no alteration of the cell morphology (Additional file 1: Figure SI11). Second, similar experiments were realized at higher concentrations of SLNs ([sorafenib] = 120 and at 84 μM for SLN^−^ and SLN^+^, respectively) and compared to free sorafenib at its limit of solubility (concentration of 5 μM). In these conditions, both SLNs exhibit a strong cytotoxic effect on the four cancer cell lines as shown in Fig. 5. As revealed on the phase contrast microscopy images, cell debris was observed for both SLN^−^ (Fig. 5C1–4) and SLN^+^ (Fig. 5D1–4) attesting cell death, whereas cells remain alive in the case of free sorafenib (Fig. 5B1–4). It is worth noting that strong cytotoxic effects for both SLNs were observed in the case of luminal B breast cancer cells (Fig. 5C1–2 and D1–2). Such an antitumoral effect, which was not reported so before, open new possible therapeutic application of sorafenib thanks to the SLNs.

## Conclusion

We report a novel approach based on nucleolipids allowing the efficient encapsulation and delivery of sorafenib. Our investigations demonstrate the formation of two types of solid lipid nanoparticles (SLNs) highly loaded with sorafenib. These SLNs, which show either negative or positive zeta potentials values, feature parallelepiped shapes. As revealed by the DLS and HPLC studies, the stability of the SLNs can be modulated depending on the nucleolipid used. Importantly, both SLN^+^ and SLN^−^ formulations are able to enhance dramatically the water solubility of sorafenib (concentrations higher than 120 μM). Such SLNs exhibit better antitumoral activities on four cancer cell lines (liver and breast cancers) compared to free sorafenib, which is limited due to its lack of solubility in water. Contrast phase microscopy images, recorded on the four cancer cell lines, exhibit a drastically cell mortality when incubated with 120 μM of SLN^−^ or 84 μM of SLN^+^. Hence, this drug could be used as the new therapeutic options in the case of liver cancers (use of sorafenib in intra-arterial chemotherapy, for example) or breast cancers. To our knowledge, this report is the first example of a study using sorafenib against luminal B breast cancers demonstrating the usefulness of the SLN approach. Altogether, the results reported in this contribution highlight the potential of nucleoside-lipid-based SLNs as drug delivery systems.

## Additional file

Additional file 1: DLS data, zeta potentials, phase contrast microscopy, and TEM of SLNs synthetized in different experimental conditions, UHPLC dosages. (PDF 14710 kb)

## Abbreviations

AFNH_4_: Ammonium formate
DLS: Dynamic light scattering
FA: Formic acid
HCC: Hepatocellular carcinoma
HPLC: High-performance liquid chromatography
LNs: Lipid nanoparticles
MeOH: Methanol
PDI: Polydispersity index
RCC: Renal cell carcinoma
SLNs: Solid lipid nanoparticles
UHPLC: Ultra high-performance liquid chromatography
