# Supplementary material for: Nucleoside-Lipid-Based Nanocarriers for Sorafenib Delivery
Source: Nanoscale Res Lett. 2018 Jan 11;13:17. doi: 10.1186/s11671-017-2420-2 (PMC5764907; doi:10.1186/s11671-017-2420-2)
Supplement: Additional file 1: — DLS data, zeta potentials, phase contrast microscopy, and TEM of SLNs synthetized in different experimental conditions, UHPLC dosages. (PDF 14710 kb) [file 11671_2017_2420_MOESM1_ESM.pdf]

Nucleoside-Lipid-Based Nanocarriers for Sorafenib Delivery

Sebastien Benizri,[a,b,c] Ludivine Ferey,[a,b,c] Bruno Alies,[a,b,c] Naila Mebarek,[a,b,c] Gaelle Vacher,[a,b,c] Ananda Appavoo,[a,b,] Cathy Staedel,[a,b,c] Karen Gaudin,[a,b,c] and Philippe Barthélémy[a,b,c]*

[a]Univ. Bordeaux, ARNA laboratory, F-33000 Bordeaux, France.

[b]INSERM, U1212, ARNA laboratory, F-33000 Bordeaux, France.

[c]CNRS, UMR 5320, ARNA laboratory, F-33000 Bordeaux, France.

* Correspondence: Philippe Barthélémy

ARNA Laboratory, team ChemBioPharm, U1212 INSERM - UMR 5320 CNRS, 146 rue Léo Saignat 33076 Bordeaux Cedex, France.

Tel +33 (0)5 57 57 48 53

Email philippe.barthelemy@inserm.fr

Supporting Information

HPLC analysis of SLNs

A reversed phase UHPLC method was developed to study SLN composition and chemical stability.

The separation was carried out with the column Syncronis C18 50 x 2.1 mm, 1.7 µm. The mobile phase consisted of 70/30 MeOH/26.5 mM ammonium acetate (pH = 7.4) (A) and 25 mM ammonium acetate in MeOH (pH 7.9) (B). A flow rate of 0.2 mL/min was used and the gradient profile was 0-2 min, 0-100% B; 2-20 min, 100% B. The column temperature was set

at 25°C. The detection was performed at 267 nm for Sorafenib and $diC_{16}dT$, and 257 nm for DOTAU. The injected volume was 1.0 µL leading to quantitation limits of 0.6 ng for Sorafenib and 15 ng for both nucleolipids, DOTAU and $diC_{16}dT$.

Standard curves for Sorafenib, DOTAU, and $diC_{16}dT$ in ethanol are shown in Figures SI1, SI2, and SI3, respectively. The HPLC analyses of both formulations are presented in Figure SI4.

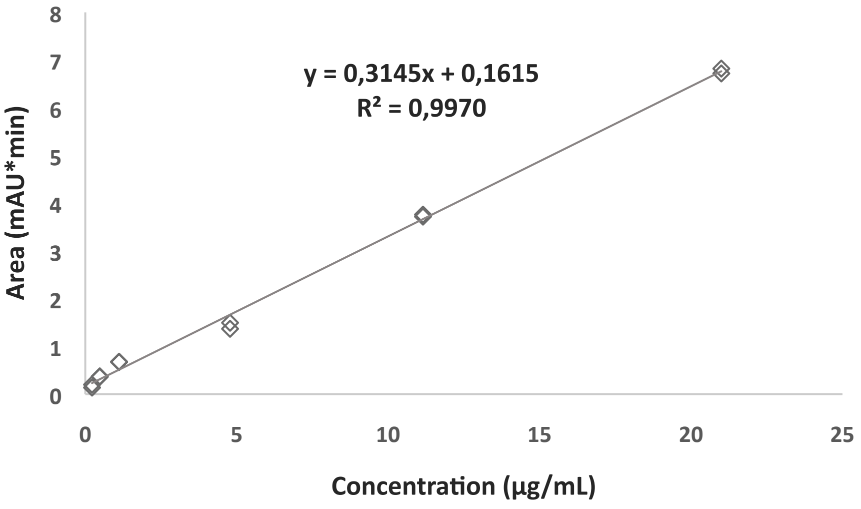

Figure SI1 Standard curve Sorafenib: A = f(C) (UV 267 nm)

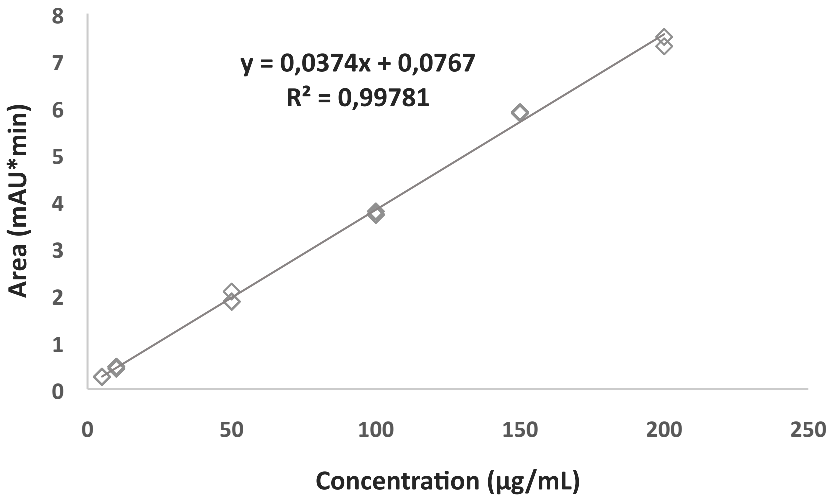

Figure SI2 Standard curve DOTAU: A = f(C) (UV 257 nm)

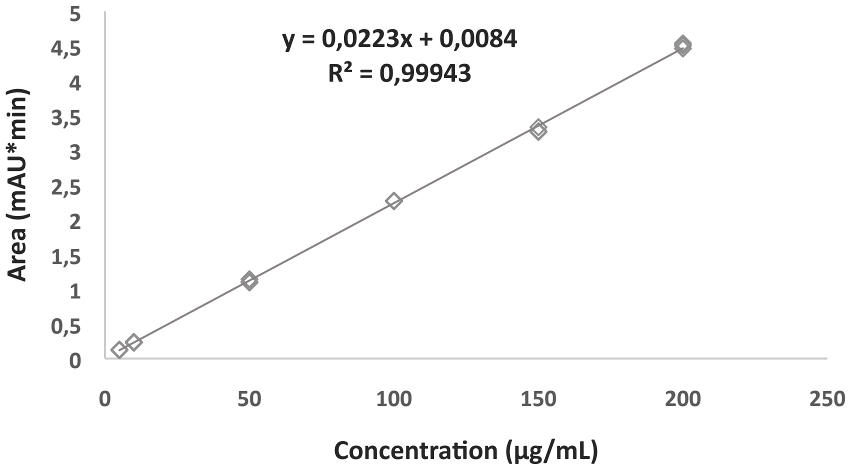

Figure SI3 Standard curve diC$_{16}$dT: A = f(C) (UV 267 nm)

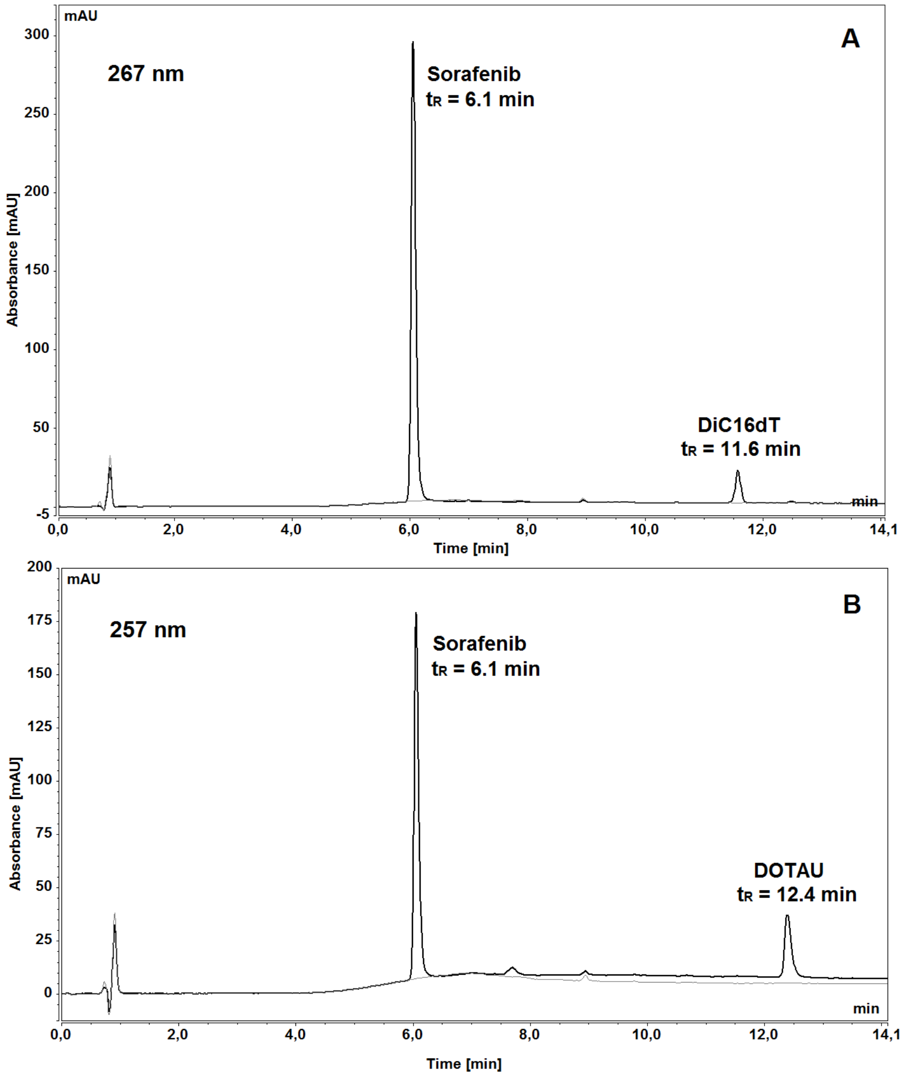

Figure SI4. Simultaneous analysis of Sorafenib and nucleolipids: diC$_{16}$dT (A) and DOTAU (B) in formulations.

Stability studies of Sorafenib/diC16dT formulations

A similar chemical stability study was carried out on Sorafenib and diC$_{16}$dT in SLNs at 4°C and 37°C. As shown on figure SI5, Sorafenib and diC$_{16}$dT remain stable over a period of one month at both temperatures.

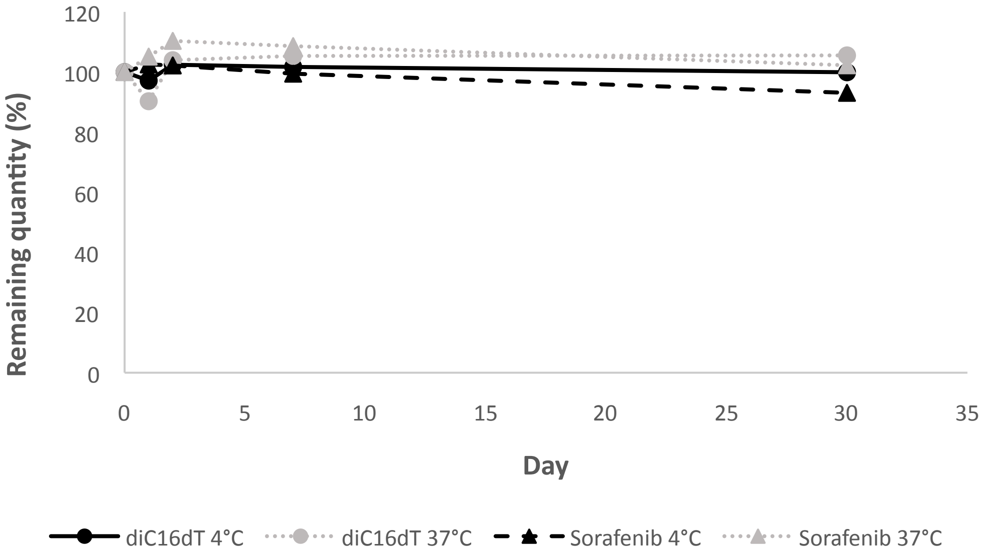

Figure SI5. Chemical stability of Sorafenib and diC$_{16}$dT in SLNs versus time at 4°C and 37°C.

The influence of dissolution solvent and temperature on DOTAU and diC$_{16}$dT was investigated.

Then, the impact of the nature of dissolution solvent was investigated. Figure SI6 A & B presents the stability of DOTAU and diC$_{16}$dT, respectively, in various solvents at 40°C. DOTAU was significantly more stable in ACN (only 10% of decrease over 3 days) than in H_2O (50% of decrease over 3 days), MeOH (up to 90% of decrease over 3 days) or EtOH (up to 85% of decrease over only 1 day) (Figure SI6.A). On the contrary, diC16dT appeared stable over time whatever the solvent nature (Figure SI6.B).

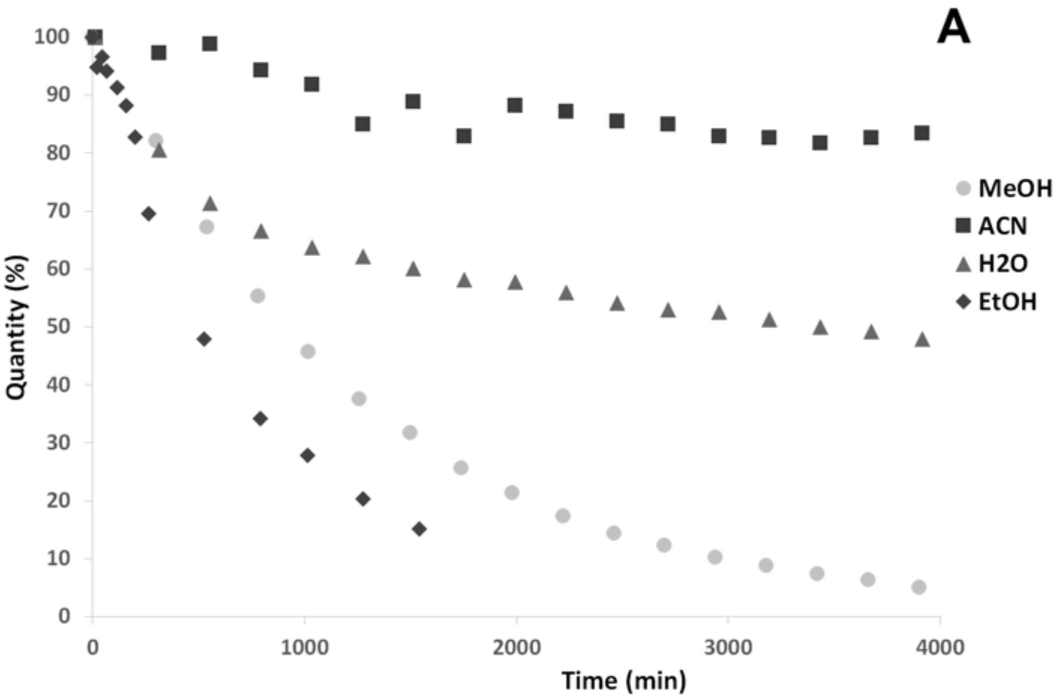

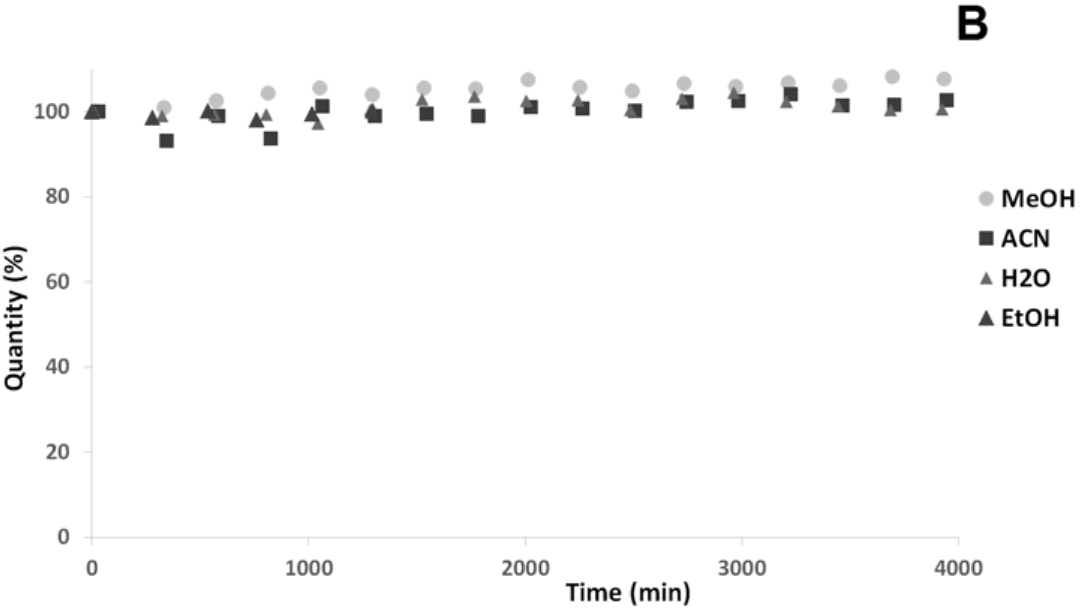

Figure SI6. Chemical studies of DOTAU (A) and diC16dT (B) at 40°C in different solvents during time**.**

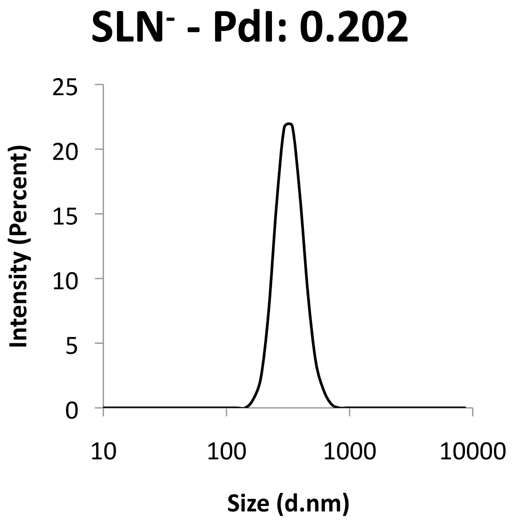

Figure SI7. Average Size of SLN$^-$ measure by DLS

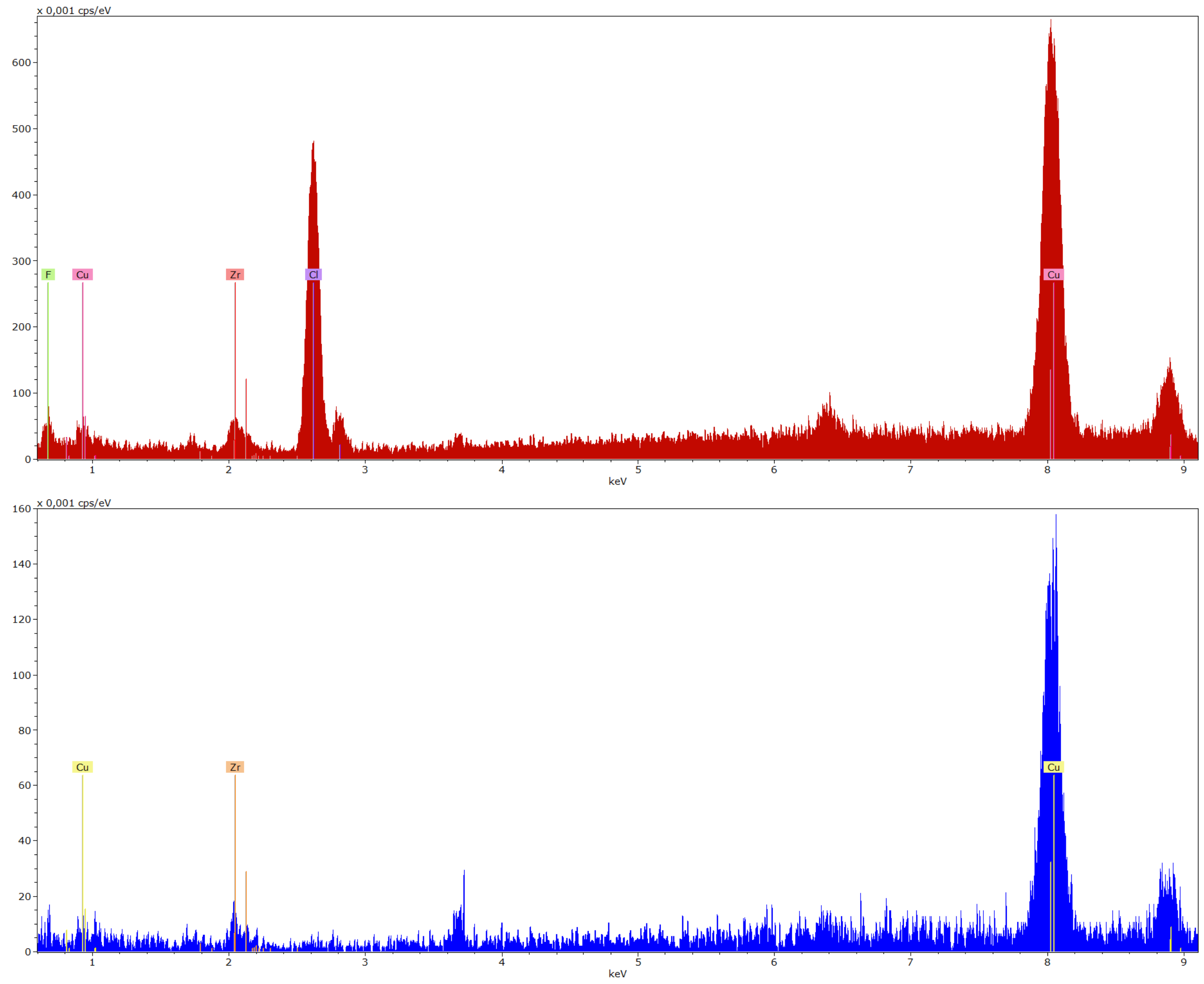

Figure SI8. EDX spectra at SLN- (up) and on the copper grid (down)

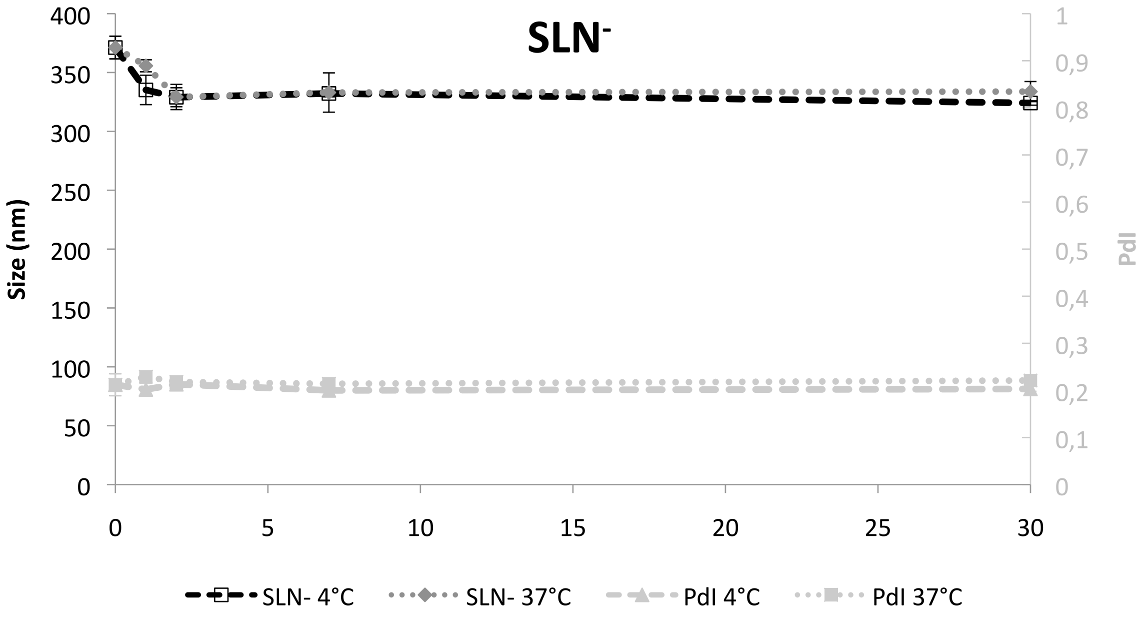

Figure SI9. Colloidal stability of SLN$^-$ measuring by DLS experiments

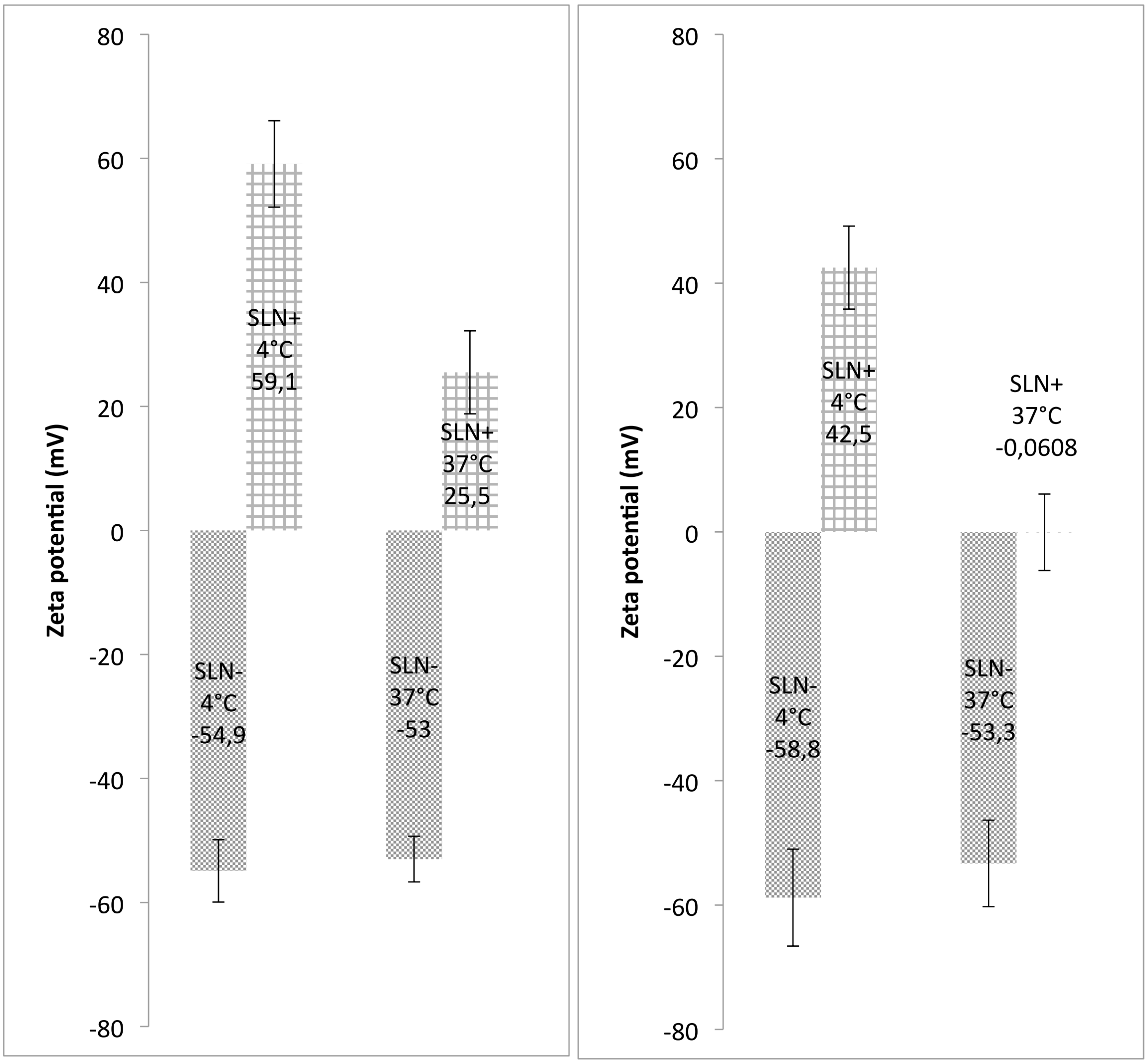

Figure SI10. Zeta potential after 7 days (left) and 30 days (right) at 4°C and 37°C for SLN^+ (up) and SLN^- (down)

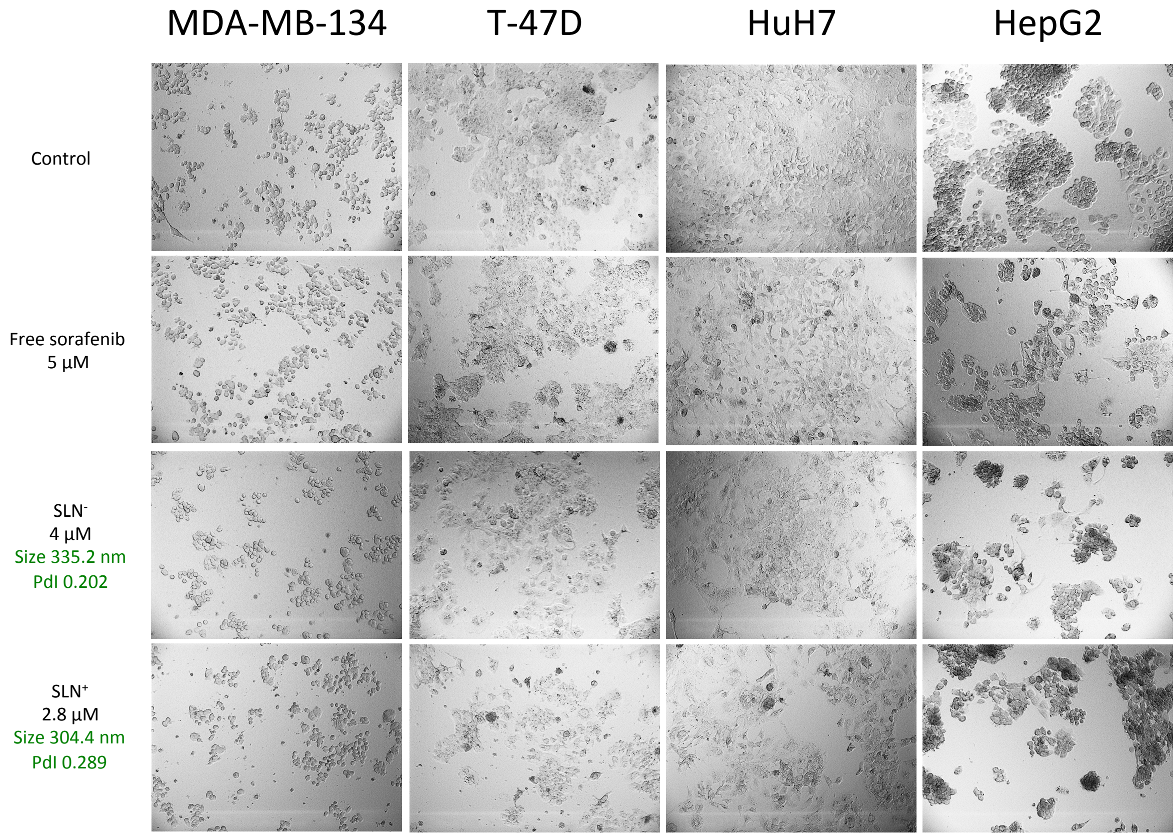

Figure SI11. Cell morphology assessed by phase contrast microscopy on carcinoma cell lines in the absence of Sorafenib (control), with 5 μM of free Sorafenib, with SLN^{-} at 4 μM and SLN^{+} at 2.8 μM upon 4 days of treatment.

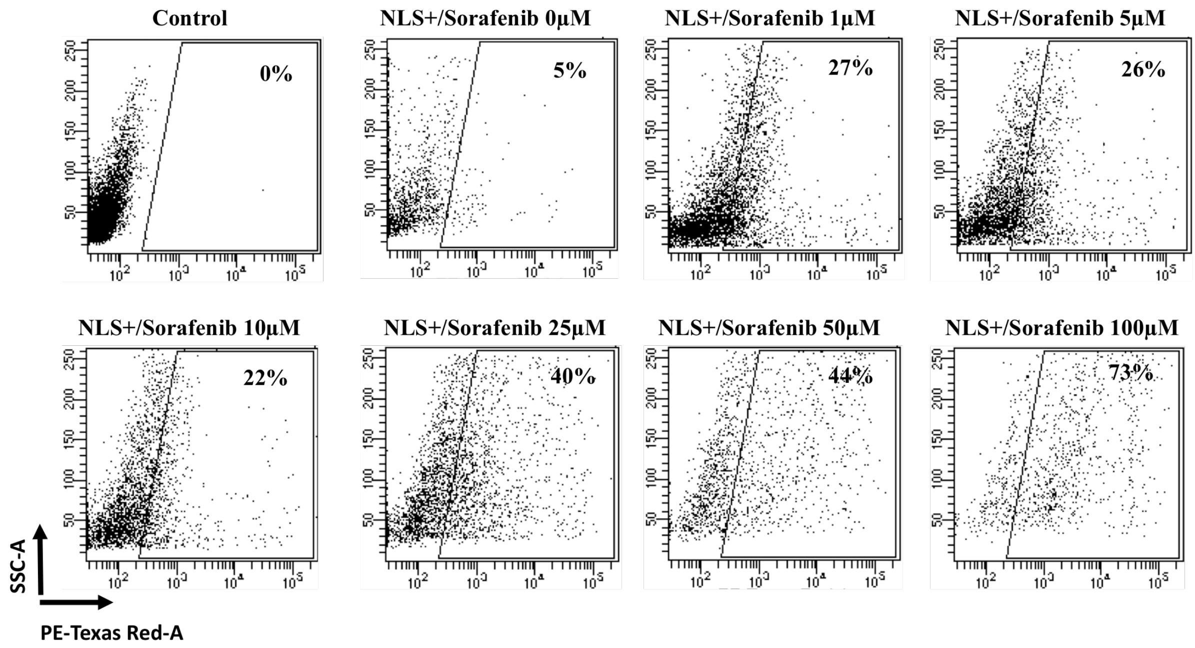

Figure SI12. Representative dot plots of cell granulosity (SSC-A) against PE-Texas Red-A detection after 4 days of HuH7 incubation with SLN+ loaded with Sorafenib at different concentrations (0, 1, 5, 10, 25, 50 and 100 µM). The percentage of PE-Texas Red positive cells were quantified by FACS. Control corresponds to HuH7 without Ethidium homodimer-1 dye.

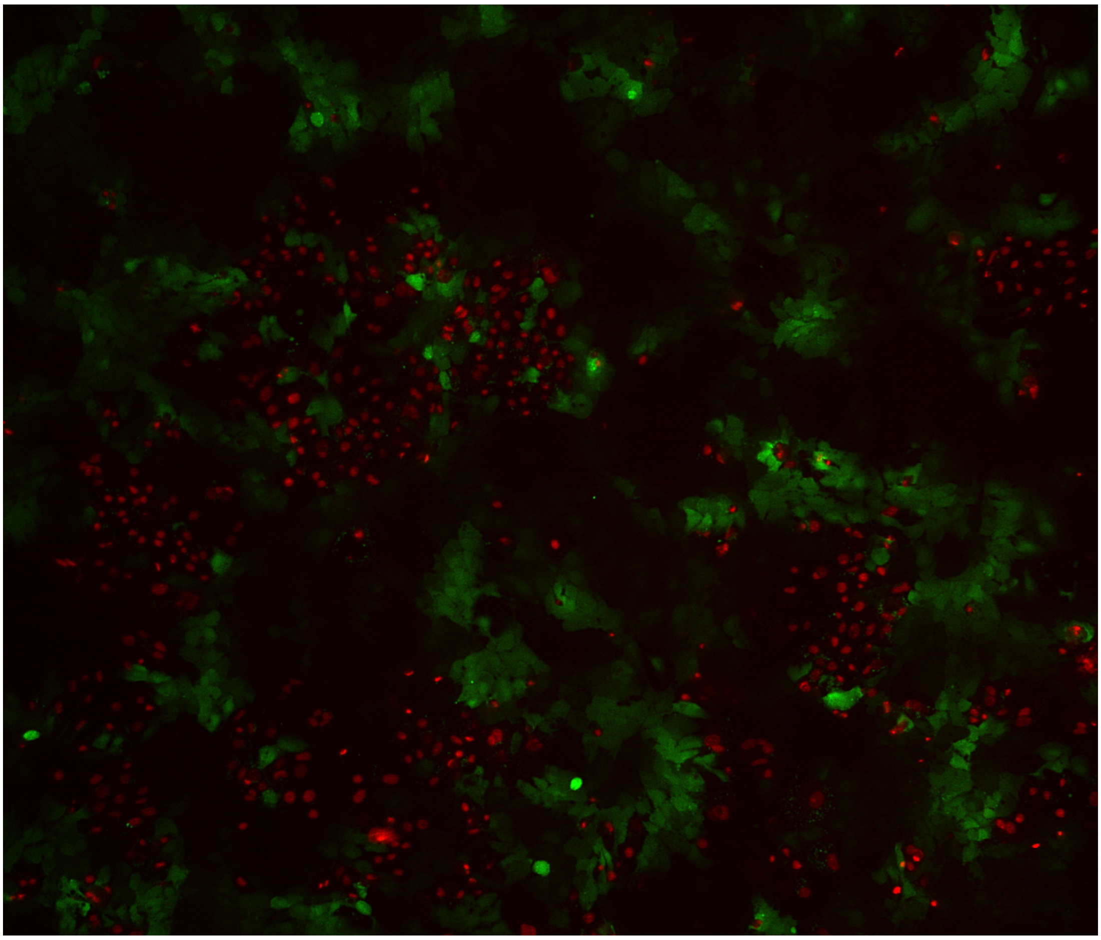

Figure SI13. HuH7 cells lines with SLN^+ at 50 μM. Calcein were used to stain live cells and ethidium stain dead cells.
